# The Mixed Blessing of Phosphor-Based White LEDs

**DOI:** 10.1289/ehp.119-a472

**Published:** 2011-11-01

**Authors:** Angela Spivey

**Affiliations:** Angela Spivey writes from North Carolina about medicine, environmental health, and personal finance.

Light-emitting diodes (LEDs), which use less energy and last longer than even compact fluorescent lights,^1^ are predicted to become the leading lighting technology in the United States as incandescent bulbs are phased out.^2^ But Abraham Haim, director of the Israeli Center for Interdisciplinary Studies in Chronobiology, will not bring white LEDs and other so-called short-wavelength lights into his home because of his concerns about their health effects. Why? Blue light such as that emitted by LEDs has been shown to suppress production of the hormone melatonin to a greater degree than other visible wavelengths emitted at the same intensity.^3^^,^^4^ Melatonin suppression has been demonstrated to disrupt sleep/wake cycles and has been linked to increased risk of breast cancer.^5^ “Modern lights . . . that use the wavelength in the range of 460 nm to 500 nm should be considered ‘bad light,’” Haim says.

Although the light from LEDs appears white, it consists of one strong, sharp peak of short-wavelength blue light (in the range of 460 nm) and a second, broader emission in the longer-wavelength part of the spectrum. This is achieved by fitting a blue LED with a fluorescent phosphor layer that absorbs part of the blue light and re-emits light of a longer wavelength.

Concerns about white-appearing LEDs center around nighttime exposure to blue light. Daytime skylight also is a blue-enriched, white-appearing light, explains George Brainard, director of the Lighting Research Program at Thomas Jefferson University, but this blue-light exposure is desirable for cuing the human circadian rhythm, which synchronizes with cycles of light/dark, eating, and activity. “To my knowledge,” Brainard says, “the white-appearing, blue-enriched LEDs do not pose the same sort of potential health consequences during the daytime.”

**Figure:**
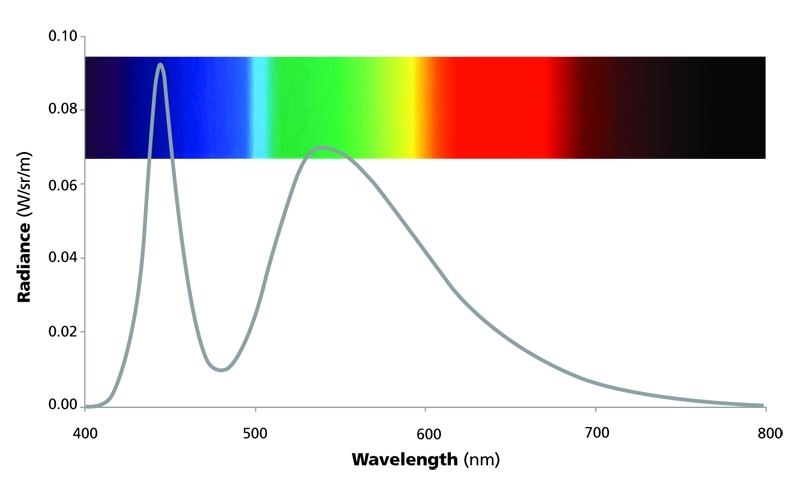
The light from white-appearing LEDs consists of one strong, sharp peak of short-wavelength light in the range of 460 nm and a second, broader emission in the longer-wavelength part of the spectrum. Ben Warfield and George Brainard/Thomas Jefferson University

You can control the light in your home, but not outdoors. That’s why Haim and other coauthors of a new study call for regulations limiting the use of certain types of lights, including LEDs, for nighttime lighting outdoors.^6^ The paper reviews research showing that nighttime exposure to white LEDs suppresses melatonin to a greater degree compared with other lighting types such as high- and low-pressure sodium, metal halide, and incandescent bulbs, and it includes measurements the researchers made of the wavelength and other spectral characteristics of several types of lights.

But the bulk of the paper consists of recommendations for reducing light pollution. In addition to limiting nighttime use of the blue-spectrum light typical of metal halide lamps and white LEDs, those suggestions include using as little light as possible outdoors, aiming for a zero increase in total outdoor lighting (for example, not adding lighting without decreasing the amount or intensity of lighting somewhere else), and prohibiting lights from being aimed upward (above the horizontal).^7^

The health problems potentially caused by current LEDs may be avoidable. “The LED industry would be wise to develop white-appearing LEDs that do not have the high emissions in the blue region of the visible spectrum for outdoor lighting applications,” Brainard says. “This would permit use of newer energy-efficient solid-state lighting while still avoiding the potential health consequences of circadian and neuroendocrine disruption from inappropriate exposure to light at night.”

“Any problems with the spectrum produced by white-light LEDs, to the extent they exist, are not inherent to LEDs themselves but rather the current implementation,” adds Jay Neitz, a professor of ophthalmology at the University of Washington Medical School. “Research is under way to improve both the spectral characteristics and efficiency of white-light LEDs. If there are problems with LEDs at the moment, they will probably be short-lived as better technologies come into use.”

Fabio Falchi, a scientist at Italy’s Light Pollution Science and Technology Institute and first author of the paper, agrees that LEDs likely will be “the future of lighting outdoors and indoors.” But he says there are ways to manage light pollution from LEDs by taking advantage of their ability to turn on and off quickly. He suggests keeping outdoor lights off or at a low level unless they are in use, which could be accomplished by using motion-detector lights that increase to full power only when a pedestrian or car approaches.

The authors also call for manufacturers to label lights with information about how much of a bulb’s light is emitted in the shorter, circadian-disrupting wavelength, much as the food industry is required to include nutritional content on labels. “We have to pay attention to several aspects of light that we are not used to paying attention to,” Falchi says.

Not all researchers agree that white LEDs pose a danger to human health. Neitz points out that the studies showing melatonin suppression from LEDs did not simulate real-world exposures. For instance, some of the studies had participants put their heads into a dome that exposed their full visual field to a single wavelength of light.^4^

**Figure:**
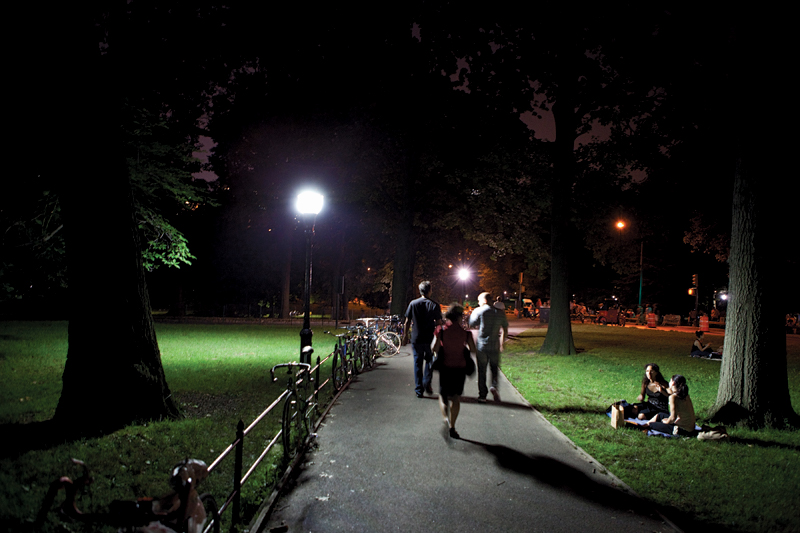
In 2009 New York City launched a pilot program to assess the ability of LED streetlights to reduce greenhouse gas emissions and improve energy efficiency. However, nighttime exposure to the blue-tinged light may have adverse effects on circadian rhythm. © Ryan Pyle/Corbis

“It’s a long stretch to go from that to make an argument about light pollution, where you are talking about light levels that would be quite low, way below where they would make a significant contribution to our circadian rhythms,” Neitz says. Real-world exposures include multiple bandwidths of light from many different sources, and in the context of all those exposures, the increased sensitivity to short-wavelength light would not make a significant difference, Neitz says.

Brainard agrees that most studies to date have not replicated real-world exposures. But he says it’s too soon to make firm conclusions because knowledge about the health effects of light is constantly evolving. For example, the first study to show melatonin suppression from any type of light used an intensity of 2,500 lux, but more recent studies have shown suppression with less than 1 lux.^8^ “Back in the 1990s no one ever imagined you could suppress melatonin in humans with less than one lux,” Brainard says. “The regulation of human neuroendocrine, circadian, and neurobehavioral responses by light, and the potential health consequences of that regulation, are going to be far more complicated and nuanced than anyone ever dreamed.”
